# Association between kidney function and incidence of dementia: 10-year follow-up of the Whitehall II cohort study

**DOI:** 10.1093/ageing/afab259

**Published:** 2022-01-17

**Authors:** Archana Singh-Manoux, Amina Oumarou-Ibrahim, Marcos D Machado-Fragua, Julien Dumurgier, Erics J Brunner, Mika Kivimaki, Aurore Fayosse, Sèverine Sabia

**Affiliations:** Université de Paris, Inserm U1153, Epidemiology of Ageing and Neurodegenerative diseases, Paris, France; Department of Epidemiology and Public Health, University College London, London, UK; Université de Paris, Inserm U1153, Epidemiology of Ageing and Neurodegenerative diseases, Paris, France; Université de Paris, Inserm U1153, Epidemiology of Ageing and Neurodegenerative diseases, Paris, France; Université de Paris, Inserm U1153, Epidemiology of Ageing and Neurodegenerative diseases, Paris, France; Cognitive Neurology Center, Lariboisière – Fernand Widal Hospital, AP-HP, Université de Paris, Paris, France; Department of Epidemiology and Public Health, University College London, London, UK; Department of Epidemiology and Public Health, University College London, London, UK; Helsinki Institute of Life Sciences, University of Helsinki, Helsinki, Finland; Université de Paris, Inserm U1153, Epidemiology of Ageing and Neurodegenerative diseases, Paris, France; Université de Paris, Inserm U1153, Epidemiology of Ageing and Neurodegenerative diseases, Paris, France; Department of Epidemiology and Public Health, University College London, London, UK

**Keywords:** dementia, chronic kidney disease (CKD), ageing, estimated Glomerular Filtration Rate (eGFR), cohort study, older people

## Abstract

**Background:**

Cognitive dysfunction is common in haemodialysis patients but whether poor kidney function in the general population is also associated with higher risk of dementia remains unclear.

**Objective:**

To examine the association of kidney function with incident dementia in community dwelling older adults.

**Design:**

Whitehall II prospective study.

**Setting:**

Population-based study on 6,050 adults, mean age 65.8 in 2007–2009.

**Methods:**

Poor kidney function, defined as estimated Glomerular Filtration Rate (eGFR) <60 ml/min/1.73 m2 in 2007–2009, and adverse change in eGFR was defined as decrease ≥4 ml/min/1.73 m2 between 2007–2009 and 2012–2013.

Incident dementia was ascertained through linkage to electronic health records, and Cox regression was used to examine associations with dementia.

**Results:**

A total of 306 cases of dementia were recorded over a mean follow-up of 10 years. Baseline eGFR <60 was associated with a hazard ratio (HR) for dementia of 1.37 (95% CI 1.02, 1.85) in analysis adjusted for sociodemographic factors, hypertension, obesity, stroke, diabetes and cardiovascular disease/medication. Removing stroke cases at baseline and censoring them over the follow-up yielded an HR of 1.42 (95% CI 1.00, 2.00) for the association between CKD and dementia. Decline of eGFR ≥4 between 2007–2009 and 2012–2013 was associated with incidence of dementia over a 6.3 year mean follow-up (HR: 1.37; 95% CI 1.02, 1.85), with somewhat stronger associations when analyses were restricted to those with eGFR ≥60 in 2007–2009 (1.56; 95% CI: 1.12, 2.19).

**Conclusion:**

Poor and declining kidney function in older adults is associated with a higher risk of dementia that is not attributable to stroke and persists after accounting for major cardiometabolic conditions.

## Key points

- It is known that cognitive impairment is common in haemodialysis patients with end-stage renal disease.
- We found poor kidney function (estimated Glomerular Filtration Rate (eGFR) <60) to be associated with a higher incidence of dementia over a 10-year follow-up.
- The association of poor kidney function with dementia was not due to major cardiometabolic diseases or stroke.

## Introduction

Cognitive impairment is common in haemodialysis patients with end-stage renal disease [1–3]; there is also a graded association between severity of chronic kidney disease (CKD) and cognitive impairment [3]. The prevalence of impaired cognitive function in those with end-stage disease and CKD is greater than that in age-matched peers [4, 5], suggesting that the association may not be simply due to age-related increase in both cognitive [6] and kidney dysfunction [7]. The kidneys and the brain, both being end organs, are thought to be susceptible to vascular damage due to similar anatomic and hemodynamic features [8].

Ageing of populations along with increasing prevalence of hypertension, diabetes and obesity has led to an increase in the prevalence of CKD worldwide [7], and there is a consensus on the need to better understand its impact on health in the general population using non-invasive measures of kidney function such as estimated Glomerular Filtration Rate (eGFR) [9]. The association between eGFR and dementia remains poorly understood [10, 11]. Early studies in this domain were mostly cross-sectional, often based on haemodialysis patients, on small numbers, and had simple measures of cognitive function, such as the Mini-Mental State Examination (MMSE). More recent reviews [5, 12–14] and meta-analyses [13, 14] suggest poor kidney function is associated with cognitive impairment and cognitive decline although most studies used a short follow-up (<10 years). The evidence for dementia is less consistent, with one study suggesting an association only with decline in kidney function and subsequent dementia over a 3-year follow-up [15], and the other showing associations with dementia to be attributable to stroke [16]. It also remains unclear whether the eGFR threshold (60 ml/min/1.73m^2^) used for CKD is also relevant for risk of dementia.

In order to resolve these inconsistencies, we examined the association of eGFR with dementia using data over 10 years in a large community dwelling, prospective cohort study. In addition to baseline eGFR, we investigated change in eGFR in relation to incidence of dementia and whether the association of CKD and dementia is attributable to stroke as postulated previously [16].

## Methods

### Study population

Whitehall II is an ongoing cohort study established in 1985–1988 among 10,308 persons (6,895 men and 3,413 women, aged 35–55 years) employed in London-based government departments [17]. Since baseline, follow-up clinical examinations have taken place approximately every 4 to 5 years. Baseline for the current analyses was the 2007–2009 wave when kidney function was first measured in the study [18], repeated in 2012–2013. In addition to data collection within the study, data over the follow-up were available using linkage to electronic health records of the UK National Health Service (NHS) for participants recruited to the study. The NHS provides most of the health care in the country, including in- and out-patient care, and record linkage is undertaken using a unique NHS identifier held by all UK residents. Written informed consent from participants and research ethics approvals were renewed at each contact; the most recent approval was from the University College London Hospital Committee on the Ethics of Human Research, reference number 85/0938.

### Exposure

We used the isotope-dilution mass spectrometry (IDMS)-traceable CKD-EPI (CKD Epidemiology Collaboration) equation to calculate eGFR [19], based on serum creatinine, age and sex. The CKD-EPI equation, expressed as a single equation, is as follows:

$$\begin{eqnarray*} eGFR\!\!\!\!\!\!\!\!\!&&=141\ast \mathit{\min}{\left( Scr/\kappa, 1\right)}^{\alpha}\ast \mathit{\max}{\left( Scr/\kappa, 1\right)}^{-1.209}\nonumber\\ &&\quad \ast\, {0.993}^{Age}\ast 1.018\ \left[ if\ female\right]\nonumber\\ &&\quad \ast\, 1.159\ \left[ if\ non- Caucasian\right] \end{eqnarray*}$$

where Scr is serum creatinine (mg/dl), κ is 0.7 for females and 0.9 for males, α is −0.329 for females and − 0.411 for males, min indicates the minimum of Scr/κ or 1 and max indicates the maximum of Scr/κ or 1.

### Outcome

Dementia cases were ascertained by linkage to three national registers (HES, the Mental Health Services Data Set and the mortality register) up to the 31 March 2019. All-cause dementia was identified based on ICD-10 codes F00-F03, F05.1, G30 and G31. The sensitivity and specificity of dementia in HES data are 78.0% and 92.0% [20]. The sensitivity in our study is likely to be further improved due to use of Mental Health Services Data Set, a national database that contains information on dementia for persons in contact with mental health services in hospitals, outpatient clinics and the community [21]. Cause-specific mortality data were drawn from the NHS national mortality register. Date of dementia was set at the first record of dementia diagnosis using all three databases.

### Covariates

#### Sociodemographic factors

Sociodemographic factors included age, sex, ethnicity (Caucasian, other), education (partial secondary school or lower, secondary school, university and higher degree) and marital status (married/cohabiting, others).

#### Obesity

Height and weight were measured at the clinical examination and BMI calculated as weight divided by height squared; BMI ≥30 kg/m^2^ was used to denote obesity [22].

#### Hypertension

The mean of two measures of systolic and diastolic blood pressure, measured in the sitting position after 5 min of rest, was used in the analysis. Hypertension was defined as systolic blood pressure ≥140 mmHg, or diastolic blood pressure ≥90 mmHg or use of antihypertensive medication.

#### Diabetes

Diagnosis was based on fasting glucose ≥7.0 mmol/l (126 mg/dl), reported physician-diagnosed diabetes, use of diabetes medication or HES record (ICD-10 codeE11).

#### CHD

CHD included ischaemic heart disease, myocardial infarction and definite angina. The ascertainment was based on ICD-10 codes I20–I25 using HESdata.

#### Stroke

Cases were defined using ICD-10 codes I60–I64 from HES records and self-reported stroke, validated against medical records [23].

#### CVD medication

CVD medication included reported use of diuretics, beta blockers, ace inhibitors, calcium channel blockers, anti-hypertensives, lipid lowering, nitrates and antiplatelets.

#### APOE genotyping

DNA was extracted from whole blood samples; two TaqMan assays (Rs429358 and Rs7412, Assay-On-Demand, Applied Biosystems) were used and run on a 7900HT analyser (Applied Biosystems) and genotypes indicated by the Sequence Detection Software version 2.0 (Applied Biosystems). APOE was modelled as those with and without ε4 allele(s).

### Statistical analysis

Characteristics of participants at the 2007–2009 wave of data collection as a function of dementia status at the end of the follow-up were examined using Student’s t-test and Chi-squared test. The shape of the association between the continuous measure of eGFR in 2007–2006 and subsequent dementia was plotted using restricted cubic splines with Harrell knots [24], using the command *mkspline* in Stata to confirm the proposed threshold of eGFR <60 to define poor kidney function [9].

We used Cox proportional hazard regression to examine the association of kidney function in 2007–2009 and subsequent incidence of dementia. Participants were followed to the date of record of dementia, death or 31 March 2019, whichever came first. Censoring participants who died over the follow-up at date of death allowed us to account for competing risk of death [25]. The analyses were first adjusted for covariates at baseline and then for these covariates treated as time-varying variables. The association of decline in eGFR between 2007–2009 and 2012–2013 with subsequent incidence of dementia was also examined using Cox regression. The start of the follow-up in these analyses was the date of the second measure of eGFR. In further analysis, the analytic sample was restricted to those with eGFR ≥60 in 2007–2009.

#### Additional analyses

Data on APOE were available on 83.1% (*N* = 5,616) of the baseline sample. In order to test the robustness of associations with dementia we reanalysed the data which also included APOE ε4 as a covariate. A recent paper from the Rotterdam study [16] concluded that impaired kidney function is not associated with dementia and that the previously suggested association might be due to higher risk of stroke among those with poor kidney function. They reached this conclusion using a method where individuals who developed incident stroke in the dementia-analyses were censored at date of stroke onset when dementia was the outcome. We also used this method to assess whether the association between poor kidney function and dementia was explained by stroke.

## Results

A total of 6,761 individuals participated in 2007–2009, among whom 536 responded to the questionnaire but did not undergo clinical examination. The analyses for incident dementia were based on 6,050 participants with 11 exclusions due to prevalent dementia and 700 due to missing data. A total of 306 cases of dementia were recorded in these individuals over a mean follow-up of 10.0 (SD 1.9) years, mean age at diagnosis was 78.1 (SD 5.3) years.

**Figure 1:**
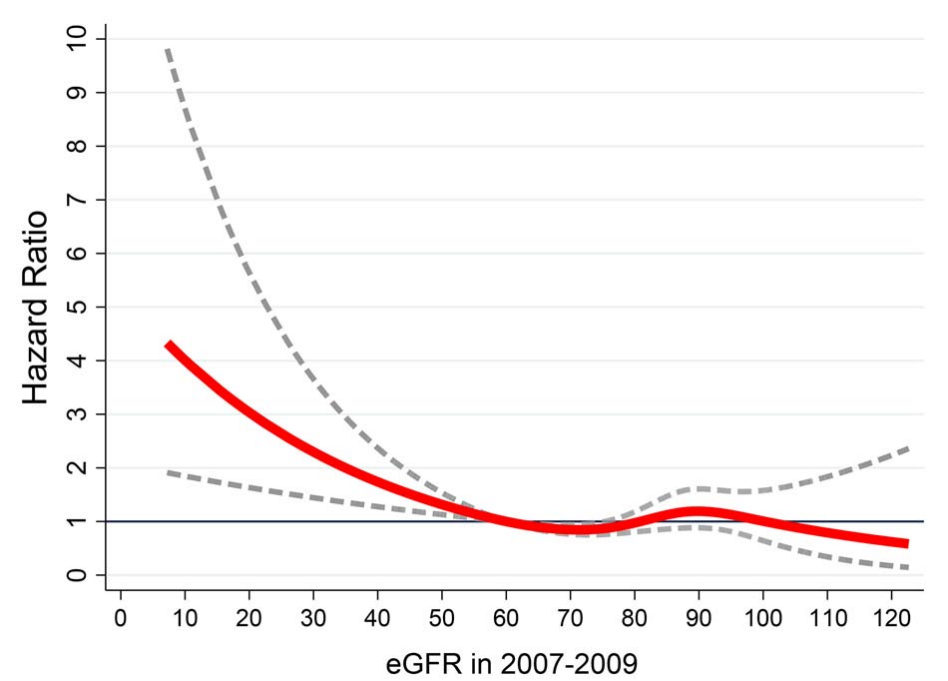
Association between continuous measure of eGFR in 2007–2009 and subsequent risk of dementia. Reference at eGFR = 60; the figure was produced using restricted cubic spline (4 knots). Analysis adjusted for age, sex, education, ethnicity, marital status, and birth-cohort (5-year groups).

The characteristics of participants at the start of the follow-up (2007–2009) as a function of dementia status at the end of the follow-up (2019) are shown in Table 1. Mean eGFR was 80.4 (SD 14.3) and participants who were diagnosed with dementia over the follow-up were older at baseline (71.3 (SD 4.9 years) compared with 65.5 (SD 5.8) years); they also had a worse sociodemographic and health profile at baseline. Analysis of continuous measure of eGFR suggested an association with dementia for eGFR <60 (Figure 1), with progressively stronger associations at lower eGFR levels. The threshold of eGFR <60 was used in subsequent analyses to designate poor kidney function.

**Table 1:** Baseline characteristics (2007–2009) according to dementia status at the end of the follow-up

	Total (*n* = 6,050)	Incident dementia (*n* = 306)	No dementia (*n* = 5,744)	*P*
CKD (eGFR <60 ml/min/1.73 m^2^), N (%)	515 (8.5)	56 (18.3)	459 (8.0)	<0.001
eGFR M (SD)	80.4 (14.3)	75.4 (16.3)	80.7 (14.1)	<0.001
Age, M (SD)	65.8 (5.9)	71.3 (4.9)	65.5 (5.8)	<0.001
Male sex, N (%)	4,344 (71.8)	206 (67.3)	4,138 (72.0)	0.074
Ethnicity, White, N (%)	5,575 (92.1)	266 (86.9)	5,309 (92.4)	<0.001
Married/cohabiting, N (%)	4,547 (75.2)	207 (67.6)	4,340 (75.6)	0.002
Education				
Low, N (%)	2,555 (42.2)	165 (53.9)	2,390 (41.6)	
Intermediate, N (%)	1,642 (27.1)	67 (21.9)	1,575 (27.4)	
High, N (%)	1853 (30.6)	74 (24.2)	1779 (31.0)	<0.001
Obesity, N (%)	1,172 (19.4)	69 (22.5)	1,103 (19.2)	0.15
Hypertension, N (%)	2,796 (46.2)	183 (59.8)	2,613 (45.5)	<0.001
Diabetes, N (%)	687 (11.4)	68 (22.2)	619 (10.8)	<0.001
Coronary Heart Disease, N (%)	800 (13.2)	65 (21.2)	735 (12.8)	<0.001
Stroke, N (%)	70 (1.2)	8 (2.6)	62 (1.1)	0.014
APOE ε4 carrier,^*^ N (%)	1,455 (27.4)	120 (45.1)	1,335 (26.5)	<0.001
CVD medication^†^, N (%)	3,127 (51.7)	213 (69.6)	2,914 (50.7)	<0.001

Abbreviations: M: Mean, SD: Standard deviation.

^*^Data on APOE missing for 738 participants.

^†^CVD medication includes diuretics, beta blockers, ace inhibitors, calcium channel blockers, anti-hypertensives, lipid lowering, nitrates and antiplatelets.

The association between poor kidney function at baseline (2007–2009) and incidence of dementia is shown in Table 2. In analyses adjusted for sociodemographic covariates (Model 1), the HR for dementia in those with eGFR <60 was 1.47 (95% CI: 1.09, 1.99). No individual covariate explained this association but inclusion of all covariates reduced the HR to 1.37 (95% CI: 1.01, 1.85). The inclusion of APOE genotype led to similar estimates. Use of time-varying covariates attenuated association to a lesser extent, suggesting that late-life risk factors have less of an impact on the risk of dementia.

**Table 2:** Association of CKD at baseline (2007–2009) with incidence of dementia.^*^

	Covariates at baseline	Time-varying covariates
	HR (95% CI)	HR (95% CI)
eGFR ≥60 ml/min/1.73 m^2^	Ref.	Ref.
CKD, eGFR <60 ml/min/1.73 m^2^ (Model 1)	1.47 (1.09, 1.99)	1.47 (1.09, 1.99)
Model 1 + Obesity	1.45 (1.07, 1.95)	1.46 (1.09, 1.98)
Model 1 + Hypertension	1.44 (1.07, 1.95)	1.46 (1.08, 1.97)
Model 1 + Diabetes	1.40 (1.04, 1.89)	1.41 (1.05, 1.91)
Model 1 + CHD	1.46 (1.08, 1.97)	1.47 (1.09, 1.98)
Model 1 + Stroke	1.47 (1.09, 1.98)	1.46 (1.08, 1.96)
**Model 1 + all covariates** ^†^	1.37 (1.01, 1.85)	1.40 (1.04, 1.89)
**Analysis with APOE as a covariate** ^‡^		
Model 1	1.47 (1.07, 2.02)	1.47 (1.07, 2.03)
**Model 1 + all covariates** ^b^ **+ APOEe4**	1.38 (1.00, 1.92)	1.42 (1.03, 1.96)

Model 1: Analysis adjusted for age, sex, education, ethnicity and marital status.

^*^N dementia cases/N total = 306/6050.

^†^All covariates: Model1 + Obesity, Hypertension, Diabetes, Coronary Heart Disease, Stroke and CVD medication.

^‡^The analysis is based on those with APOE e4 (yes/no) data, N dementia cases/N total in these analyses = 266/5312.

The mean decline in eGFR between 2007–2009 and 2012–2013 was 1.4; we used a decline ≥4 ml/min/1.73m^2^ to designate decline in order to have sufficient numbers in analyses. Decline in eGFR was associated with a 1.39 (95% CI: 1.03, 1.86) HR of incidence of dementia over a mean follow-up of 6.3 years (SD 1.0) in analysis adjusted for sociodemographic covariates (Table 3). This association was robust to adjustment for all covariates (HR 1.37, 95% CI 1.02, 1.85), including APOE genotype (HR 1.46, 95% CI: 1.06, 2.00) and was similar when time-varying covariates were used in the analysis. Restricting these analyses to those with eGFR ≥60 ml at baseline also showed an association between decline in eGFR and incidence of dementia (Appendix, eTable 1), the fully adjusted HR was 1.59 (95% CI: 1.11, 2.28).

**Table 3:** Association of decline in eGFR (≥4 between 2007–2009 and 2012–2013) with incidence of dementia^*^

	Covariates at baseline	Time-varying covariates
	HR (95% CI)	HR (95% CI)
Decline in eGFR <4 ml/min/1.73 m^2^	Ref.	Ref.
Decline in eGFR ≥4 ml/min/1.73 m^2^ (Model 1)	1.39 (1.03, 1.86)	1.38 (1.03, 1.85)
Model 1 + Obesity	1.39 (1.04, 1.87)	1.37 (1.02, 1.84)
Model 1 + Hypertension	1.38 (1.03, 1.86)	1.38 (1.03, 1.85)
Model 1 + Diabetes	1.36 (1.01, 1.82)	1.34 (1.00, 1.80)
Model 1 + CHD	1.39 (1.03, 1.86)	1.38 (1.03, 1.85)
Model 1 + Stroke	1.38 (1.03, 1.85)	1.37 (1.02, 1.84)
**Model 1 + all covariates** ^†^	1.37 (1.02, 1.85)	1.34 (1.00, 1.80)
**Analysis with APOE as a covariate** ^‡^		
Model 1	1.44 (1.05, 1.97)	1.43 (1.04, 1.95)
**Model 1 + all covariates** ^†^ **+ APOEe4**	1.46 (1.06, 2.00)	1.42 (1.03, 1.94)

Model 1: Analysis adjusted for age, sex, education, ethnicity and marital status.

^*^N dementia cases/N total = 182/5153.

^†^All covariates: Model1 + Obesity, Hypertension, Diabetes, Coronary Heart Disease, Stroke and CVD medication.

^‡^The analysis is based on those with APOE e4 (yes/no) data, N dementia cases/N total = 160/4618.

In additional analyses participants with prevalent stroke were excluded (*N* = 70) and those with incidence of stroke over the follow-up were censored at date of stroke (*N* = 154). The association of CKD (HR 1.42 (1.00, 2.00); eTable 2) and change in eGFR (HR 1.52 (1.09, 2.12); eTable 3) with incidence of dementia in fully adjusted analyses remained similar to that in the main analysis.

## Discussion

Our study presents three key findings. One, the eGFR <60 threshold, used to denote risk of CKD, is also valid for risk of dementia. Two, we found a robust association of eGFR <60 with incidence of dementia that was not attributable to stroke and persisted after adjustment for other major cardiometabolic conditions. Three, declining eGFR, even when confined to analysis on persons with eGFR ≥60 at baseline, was associated with higher incidence of dementia. The global prevalence of CKD in the general population is estimated to be 8 to 16% [26], and it is associated with increased risk of cardiovascular disease, impaired quality of life and reduced life expectancy [27, 28]. Our findings, based on a large general population study with a long follow-up, extend evidence on the adverse outcomes associated with poor kidney function to dementia.

A meta-analysis published in 2012 found a 39% higher odds for cognitive impairment in patients with CKD [14]. The authors noted limitations such as the use of only MMSE to measure cognitive function, variability in age when association is considered and differences in covariates used in studies. A more recent meta-analysis, published in 2017, reported a 35% increased risk of cognitive impairment or dementia for albuminuria (OR 1.35, 95% CI 1.06, 1.73) but eGFR <60 did not have a robust association (OR 1.28, 95%CI 0.99, 1.65) [13], although it is worth noting that the effect size in the meta-analysis was similar for albuminuria and eGFR <60.

Eight studies [15, 29–35] were included in the 2017 meta-analysis [13]; of these only two were on dementia [15, 35], one used cognitive impairment or dementia as the outcome [32], while the other five were on cognitive decline/impairment with no measure of dementia. The longest follow-up in the studies on dementia was 5.8 years in the 3C study [15], but in this study the dementia status of participants not present at the follow-up at 2, 4 and 7 years from baseline was unknown, leading them to be excluded from the analyses. The largest of the eight studies in the meta-analysis (19,399 out of 36,636 individuals in the meta-analysis) used a six-item telephone-based interview to determine cognitive impairment [31]. Thus, our study addresses limitations in studies in this meta-analysis, which were a short follow-up, analyses of cognitive decline using simple measures to denote dementia and use of combined endpoints, often cognitive impairment and dementia.

Data from the Systolic Blood Pressure Intervention Trial (SPRINT) show eGFR <60 not to be associated with increased risk of dementia or mild cognitive impairment over a 5.1 year follow-up [36]. However, this study found higher risk of dementia/mild cognitive impairment in a small set of individuals (465 out of 9,361 in the trial) where eGFR decline was ≥30%. These results are not directly comparable to our study as the SPRINT trial enrolled participants with hypertension, and the outcome examined was probable dementia or mild cognitive impairment.

A recent publication from the Rotterdam study found impaired kidney function to be associated with a higher risk of stroke, but not dementia [16]. The use of continuous measures of kidney function (eGFR based on creatinine, cystatin-C or both) is a limitation in these analyses as it is based on the assumption that the risk of dementia is progressively higher with decreasing kidney function. Our data clearly show the risk to be confined to those with eGFR<60 (Figure 1). In our study, censoring participants who suffered a stroke over the follow-up, exactly like in the Rotterdam study, did not affect the strength of the association between poor kidney function and risk of incident dementia (Table S2).

One previous study found decline in eGFR to be associated with a 69% higher risk of dementia, but these analyses were based on less than a third (2,382 out of 7,839) of the participants with baseline eGFR [15]. Our analyses were based on over 85% (5,153 of 6,050) participants with a baseline eGFR measure. We extended the analysis of decline in eGFR to those with eGFR ≥60 at baseline. Both these analyses show that declining eGFR is associated with increased risk of dementia. While our analyses contain a smaller number of CKD patients than would be seen in an older and sicker population, the value of these results lies in potential for prevention by highlighting the association of poor kidney function, using eGFR which is a convenient non-invasive blood-based measure, and dementia in the general population.

The mechanisms linking renal dysfunction and neurocognitive disorders, such as dementia, are unclear [10, 11]. Both conditions share several risk factors such as hypertension and diabetes mellitus [28, 37], and are characterised by small vessel disease [13]. The cerebro-renal connection could be due to small vessel disease as both end organs have similar anatomical and physiologic characteristics [8]. One study found the CKD-dementia association to be independent of brain atrophy and small vessel disease [38], but the small size of that study precludes firm conclusions. Kidney dysfunction is related to small vessel disorders, including glomerular endothelium dysfunction and lipohyalinosis, a cerebral small vessel disease affecting small arteries, arterioles or capillaries in the brain. A recent review highlighted the role of interactions of uremic neurotoxins with neural progenitor cells, the brain vasculature, the glymphatic system and monoaminergic neurons [11].

The study findings need to be interpreted with its strengths and limitations. The main weakness of the study is only being able to adjust for traditional risk factors. There is robust evidence of low eGFR being associated with silent lacunar infarction, white matter lesions and cerebral microbleeds [39]. A further limitation is that participants are likely to be healthier than those with advanced kidney disease, both in terms of risk factor profiles and incidence of dementia. However, the association between risk factors and disease of interest is not necessarily affected [40] as we have previously shown the association between cardiovascular risk factors and risk of CVD to be similar to that in the general population [41]. Finally, although the follow-up in the present study is longer than in previous studies on eGFR and dementia, the follow-up is not long enough to rule out reverse causations where estimates can be biased due to the long preclinical phase of dementia affecting multiple processes.

The primary strength is use of a longitudinal design in individuals not selected for kidney disease, with repeat assessment of kidney, along with a follow-up for dementia in all participants. Use of linkage to electronic health records for dementia ascertainment has the advantage of allowing analysis on everyone recruited to the study rather than only those who continue to participate over the course of the study and are available for an in-person clinical ascertainment of dementia. In the UK, HES records on dementia have high specificity but modest sensitivity (78%) due to missing of milder cases of dementia [20]. We additionally used the UK mental health database in order to improve the sensitivity of dementia diagnosis [21]. While kidney function can be measured with multiple indicators, eGFR is seen to be a robust measure [27]. The availability of a range of covariates, including diabetes and hypertension—the main risk factors of kidney disease [27], is a further strength.

In conclusion, our data suggest a higher risk of dementia in those with eGFR <60, possibly due to common pathogenesis. The continuing increase in the prevalence of CKD, cost of care and poor outcomes makes CKD surveillance a public health priority. While cardiovascular disease and reduction in life expectancy are recognised adverse outcomes of CKD, it is possible that dementia is also part of the risk associated with CKD.

## Supplementary Material

aa-21-1294-File002_afab259
