# Supplementary material for: Association between kidney function and incidence of dementia: 10-year follow-up of the Whitehall II cohort study
Source: Age Ageing. 2022 Jan 17;51(1):afab259. doi: 10.1093/ageing/afab259 (PMC8782607; doi:10.1093/ageing/afab259)
Supplement: aa-21-1294-File002_afab259 [file aa-21-1294-file002_afab259.docx]

**Association between kidney function and incidence of dementia: 10-year follow-up of the Whitehall II cohort study**

**Appendix**

eTable 1. Association of decline in eGFR (≥4 between 2007-2009 and 2012-2013) with incidence of dementia in those with eGFR ≥60 in 2007-2009.

eTable 2. Association of CKD with incidence of dementia, stroke cases censored over the follow-up.

eTable 3. Association of decline in eGFR (decline ≥4 ml/min/1.73 m^2^ between 2007-2009 and 2012-2013) with incidence of dementia, stroke cases censored over the follow-up.

**eTable 1. Association of decline in eGFR (≥4 between 2007-2009 and 2012-2013) with incidence of dementia in those with eGFR ≥60 in 2007-2009.** ^a^

|  | **Covariates at baseline** |
| --- | --- |
|  | **HR (95% CI)** |
| Decline in eGFR ≥4 ml/min/1.73 m^2^ | Ref. |
| Decline in eGFR ≥4 ml/min/1.73 m^2^ (Model 1) | 1.57 (1.13, 2.19) |
| Model 1 + Obesity | 1.58 (1.13, 2.20) |
| Model 1 + Hypertension | 1.59 (1.14, 2.22) |
| Model 1 + Diabetes | 1.54 (1.10, 2.14) |
| Model 1 + CHD | 1.57 (1.13, 2.19) |
| **Model 1 + all covariates**^b^ | 1.56 (1.12, 2.19) |
| **Analysis with APOE as a covariate**^c^ |  |
| Model 1 | 1.57 (1.10, 2.25) |
| **Model 1 + all covariates**† **+ APOEe4** | 1.59 (1.11, 2.28) |

Model 1: Analysis adjusted for age, sex, education, ethnicity, and marital status.

^a^N dementia cases/ N total=140/4721.

^b^All covariates: Model1 + Obesity, Hypertension, Diabetes, Coronary Heart Disease, Stroke, and CVD medication.

^c^The analysis is based on those with APOE e4 (yes/no) data, N dementia cases/ N total=122/4245.

**eTable 2. Association of CKD with incidence of dementia, stroke cases censored over the follow-up.**^a^

|  | **Covariates at baseline** |  | **Time varying covariates** |
| --- | --- | --- | --- |
|  | **HR (95% CI)** |  | **HR (95% CI)** |
| eGFR ≥60 ml/min/1.73 m^2^ | Ref. |  | Ref. |
| eGFR <60 ml/min/1.73 m^2^ (Model 1) | 1.46 (1.06, 2.01) |  | 1.46 (1.06, 2.01) |
| Model 1 + Obesity | 1.44 (1.04, 1.98) |  | 1.45 (1.06, 2.00) |
| Model 1 + Hypertension | 1.44 (1.04, 1.98) |  | 1.46 (1.06, 2.01) |
| Model 1 + Diabetes | 1.38 (1.00, 1.90) |  | 1.40 (1.02, 1.93) |
| Model 1 + CHD | 1.45 (1.05, 1.99) |  | 1.46 (1.06, 2.01) |
| **Model 1 + all covariates**^b^ | 1.36 (0.98, 1.88) |  | 1.41 (1.02, 1.94) |
| **Analysis with APOE as a covariate**^c^ |  |  |  |
| Model 1 | 1.48 (1.05, 2.08) |  | 1.48 (1.05, 2.08) |
| **Model 1 + all covariates**† **+ APOEe4** | 1.42 (1.00, 2.00) |  | 1.48 (1.04, 2.09) |

N dementia cases/ N total=274/5974.

Model 1: Analysis adjusted for age, sex, education, ethnicity, and marital status.

^a^Prevalent stroke (N=70) was excluded, and stroke occurring over the follow-up (N=154) was censored at date of stroke in these analyses.

^b^All covariates: Model1 + Obesity, Hypertension, Diabetes, Coronary Heart Disease, Stroke, and CVD medication.

^c^The analysis is based on those with APOE e4 (yes/no) data, N dementia cases/ N total=238/5246.

**eTable 3. Association of decline in eGFR (decline ≥4 ml/min/1.73 m^2^ between 2007-2009 and 2012-2013) with incidence of dementia, stroke cases censored over the follow-up.**^a^

|  | **Covariates at baseline** |  | **Time varying covariates** |
| --- | --- | --- | --- |
|  | **HR (95% CI)** |  | **HR (95% CI)** |
| Decline in eGFR <4 | Ref. |  | Ref. |
| Decline in eGFR ≥4 (Model 1) | 1.48 (1.09, 2.01) |  | 1.47 (1.08, 2.01) |
| Model 1 + Obesity | 1.48 (1.09, 2.02) |  | 1.46 (1.07, 2.00) |
| Model 1 + Hypertension | 1.49 (1.09, 2.04) |  | 1.48 (1.09, 2.02) |
| Model 1 + Diabetes | 1.45 (1.06, 1.98) |  | 1.44 (1.06, 1.96) |
| Model 1 + CHD | 1.48 (1.09, 2.02) |  | 1.48 (1.09, 2.02) |
| **Model 1 + all covariates**^b^ | 1.48 (1.08, 2.02) |  | 1.45 (1.06, 1.98) |
| **Analysis with APOE as a covariate**^c^ |  |  |  |
| Model 1 | 1.49 (1.07, 2.07) |  | 1.49 (1.07, 2.06) |
| **Model 1 + all covariates**^b^ **+ APOEe4** | 1.52 (1.09, 2.12) |  | 1.48 (1.06, 2.07) |

Model 1: Analysis adjusted for age, sex, education, ethnicity, and marital status.

^a^Prevalent stroke (N=70) was excluded, and stroke occurring over the follow-up (N=92) was censored at date of stroke in these analyses. N dementia cases/ N total=163/5075.

^b^All covariates: Model1 + Obesity, Hypertension, Diabetes, Coronary Heart Disease, Stroke, and CVD medication.

^c^The analysis is based on those with APOE e4 (yes/no) data, N dementia cases/ N total=144/4551.
